# Feasibility of Extirpating the Thyroid Gland in Some Cases of Disease, with Report of Case

**Published:** 1877-07

**Authors:** J. F. Miner


BUFFALO

### Medical and Surgical Journal.

VOL XVI. JULY, 1877. No. 12.

### Original Communications.

———:o:———

ART. I.—*Feasibility of Extirpating the Thyroid Gland in some Cases of Disease, with report of Case.* By J. F. MINER, M. D.

Having my attention recently called to the feasibility or possibility of removing the thyroid gland by operation when diseased, and seeing how surgical authors have most thoroughly discouraged the attempt and driven us from the field of contest when it seems in some cases cowardice to retire and abandon all effort, I propose to call attention to the question already perhaps settled in the minds of most surgeons, and to relate a case of some interest in connection with the subject.

All are familiar with the various plans which have been proposed for removal of goitrous tumors or relief of the distresses it occasions. Ligature of one or more of the arteries supplying the gland has been made with success in a few instances. Tapping encysted goiter and injection of the cyst has been followed by relief, perhaps by cure, but is not unattended by danger. Pressure with adhesive plaster has been reported as curative of the disease. Setons have been introduced, but this is said to be attended by serious danger, admission of air to the veins having resulted where the seton has been employed. Injections of persulphate of iron in vascular and pulsating bronchocele has been tried, as has been said, with great peril and small benefit. Division of the tissues compressing the growth and the isthmus of the gland with the hope of relieving impending suffocation, has also been practiced, but mostly without satisfactory results, and to quote a paragraph from an interesting article in the *New York Medical Journal*, by S. B. St. John, M. D., who says, "The method of treating goiter by electrolysis certainly deserves a place. Its advocates, like electricians generally, admit no limit to its power." Althaus says: "All cases of bronchocele, however large, may be cured by electrolysis, if the treatment is continued for a sufficient length of time. The negative pole is introduced into the tumor, and the positive applied by a sponge-electrode in the neighborhood. The current should be passed five to fifteen minutes and two or three times a week. The action is ascribed partly to mechanical action of the nascent hydrogen in breaking up the tumor, and partly to the chemical action of the alkalies at the negative pole."

*Extirpation with the knife* is discouraged on account mainly of the liability of hemorrhage and other risks of the operation. Liston says, "The operation is attended by so absolute a certainty of fatal results as not to be warranted under any circumstances, far less for removal of deformity." Skey says, "The operation has been proposed in cases of great enlargement or of malignant disease, but is in my opinion inadmissible." Gross says, "If a surgeon should be so adventurous or foolhardy as to undertake the enterprise, I should not envy him his feelings while engaged in the performance of it. * * * * Every step he takes will be environed with difficulties, every stroke of his knife followed by a torrent of blood, and lucky will it be for him if his victim lives long enough to enable him to finish his horrid butchery. Whether, then, we view this operation in relation to the difficulties which must necessarily attend its execution or with reference to the severity of the subsequent inflammation, it is equally deserving of rebuke and condemnation, and no honest and sensible surgeon, it seems to me, would engage in it."

All this denunciation requires no reply other than relation of cases and statistics of results. Statistics collected by Dr. Welch show only twenty-five per cent. of deaths, one of these from ordinary causes such as may complicate any great operation. While, according to S. B. St. John, M. D., Gûrlt's table gives twenty cases, eighteen cures, one death, a better record than many operations whose legitimacy is fully established. Dr. Green has successfully removed goitrous tumors by a novel and original method which consists in removing the tumor regardless of hemorrhage as quickly as possible, and tying the bleeding or torn arteries as they appear after removal.

After consulting our standard authors the question still remains what is the safest and best method to pursue. Without expecting to answer this question, I will briefly report a case of cystic degeneration of the gland which resulted in perfect cure after extirpation, but have no doubt great differences are to be observed in goitrous tumors, and that no one method of procedure will be found applicable in all cases or to the various forms of the disease.

Charles Hammel, aged fifteen years, of Wilcox, Pa., consulted me July 8th, 1876, for a tumor of the right portion of the thyroid gland as I inferred mainly from its movements in deglutition, location, size, period of growth and other appearances. Parents said, "Growth was first noticed seven years ago. After severe cold throat swelled very large, as large as a large bowl. When the swelling was reduced it left a lump about the size of a walnut, which continued for several years without change; for past two years increasing in size, interfering badly with his breathing, so that he cannot take any severe exercise, and at last when asleep, his efforts to breathe are painful to see." He came to Buffalo as a last resource, his friends willing to accept all risks offering any hope of relief, as he could not longer endure the distress of suffocation. At my suggestion he was admitted to the hospital of the Sisters of Charity and situation noticed for a few days, during which period he was examined by quite a large number of surgeons who visited the hospital and were attracted by his expression of anxiety and distress. Seeing the impending death if no relief could be afforded, I decided to attempt removal, and if insuperable difficulties were met, to change the plan to meet the necessities as presented. The tumor was fully exposed upon its outer surface and its character noticed. By carefully separating it from adjacent parts we found it possible to nearly surround the growth with the fingers and handle of scalpel. A few not large arteries were ligated as they were divided or ruptured in the process of separating the tumor from its connections. The principal vascular supply was from its base, but no vessels of the size of the inferior thyroid were divided. Galvanic cautery was applied to the deep surface, which afforded profuse hemorrhage from numerous small vessels which could not be easily secured by ligature. The neck of the gland divided and right portion of gland removed attached to the tumor. Until this time I had no doubt of the goitrous character of the growth, but the comparative ease of removal, absence of dangerous hemorrhage or other difficulty, led me to inquire again if really the right position of the thyroid body had been thus removed. More careful examination showed cystic degeneration of the gland. The largest cyst containing about four ounces of viscid yellow fluid, resting upon remaining portion of gland and surrounded by several small cysts. The gland did not appear to be wholly involved in disease. The boy left the hospital in about three weeks, fully recovered and relieved of all trouble.

The whole subject of Goiter, Bronchocele, Struma, Derbyshire neck, etc., as included under the general head of benign enlargements of the thyroid gland are most thoroughly before the profession and I have no expectation of adding anything important to our knowledge of this form of disease. Goiter should be distinguised from Bronchocele, ursa, cystic tumors, etc. All are aware that Goiter has been described under an extensive variety of forms which Virchow has shown may all be reduced to, and regarded as, hypertophy or hyperplasia of the normal gland elements—that the differences arise from the different degrees of implication of the different normal elements and from secondary processes which have the character of retrograde metamorphosis. The changes are ordinarily unimportant, but when the gland is the seat of pathological conditions resulting in enlargement sufficient to endanger life by pressure upon other organs, their clinical importance is sufficiently obvious.

I may be allowed in passing to remark upon the vascular supply of the organ which is said to be far beyond the requirements of its tissues and received from four large arteries. The two superior thyroid from the external carotid and the two inferior thyroid from the thyroid axis assisted sometimes by a fifth, the median thyroid from the innominate or arch of aorta. Mayer estimates that the thyroid receives as much blood as the forearm, others that the arteries of the thyroid are eight times as large as those of the brain considered relatively to the weight of the respective organs. At all events, the organ is so vascular that incisions made into it are often attended by dangerous and even fatal hemorrhage. The vascular supply is changed by disease. It is augmented, and I mistrust it may be *lessened*,—vessels diminished in size and changed in distribution, so that a diseased gland may in some cases be more easily and safely extirpated than a healthy one.

I only desire in this paper to call attention to what I regard established, that some goitrous tumors may be extirpated with the knife, if other means fail, with *reasonable hope of success.*

